# Supplementary material for: Magnetic Core-Shell Nanoparticles Using Molecularly Imprinted Polymers for Zearalenone Determination
Source: Molecules. 2022 Nov 23;27(23):8166. doi: 10.3390/molecules27238166 (PMC9738517; doi:10.3390/molecules27238166)
Supplement: Supplementary file 1 [file molecules-27-08166-s001.zip › molecules-2008144-supplementary.pdf]

Supplementary Materials

Magnetic Core-Shell Nanoparticles Using Molecularly Imprinted Polymers for Zearalenone Determination

Luis Calahorra-Rio, Miriam Guadaño-Sánchez, Tamara Moya-Cavas and Javier Lucas Urraca *

Department of Analytical Chemistry, Faculty of Chemistry, Complutense University of Madrid, Plaza Ciencias, 2, 28040 Madrid, Spain

* Correspondence: jurracar@ucm.es

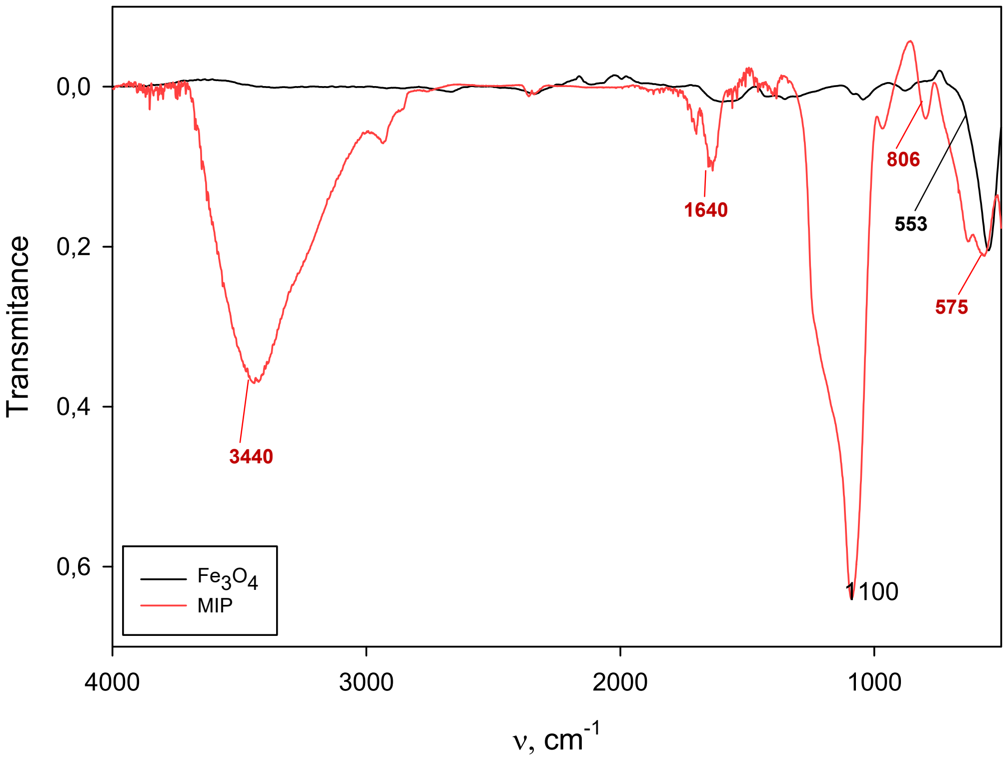

Figure S1. FT-IR spectra of Fe_3O_4 magnetic cores before (black line) and after coating, MIP (red line).

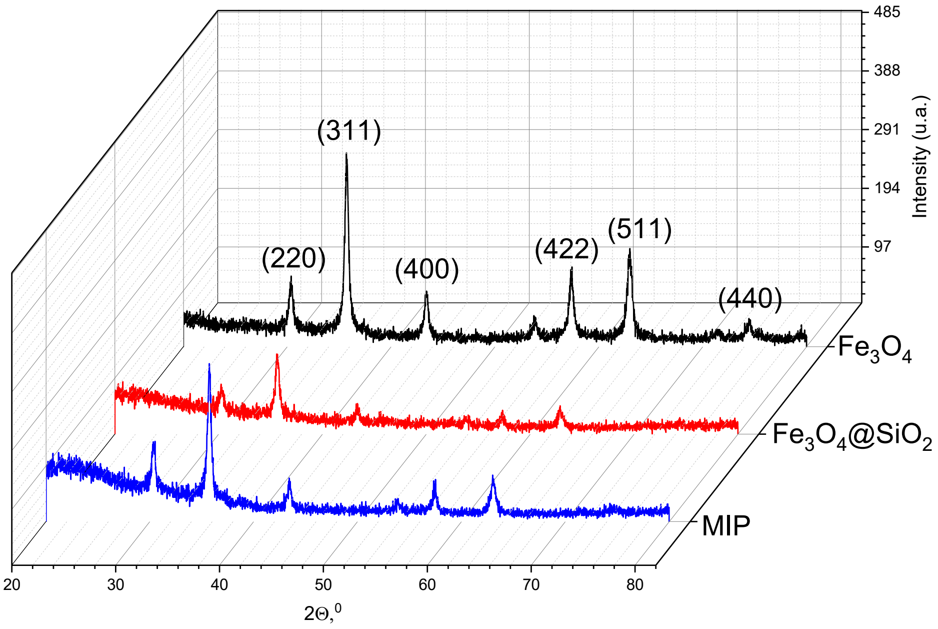

Figure S2. XRD pattern (black line) of magnetic cores before coating, (red line) of magnetic cores coated with SiO_2 and (blue line) nanocomposite of $Fe_3O_4@SiO_2$ coated with sol-gel MIP after template molecule extraction.

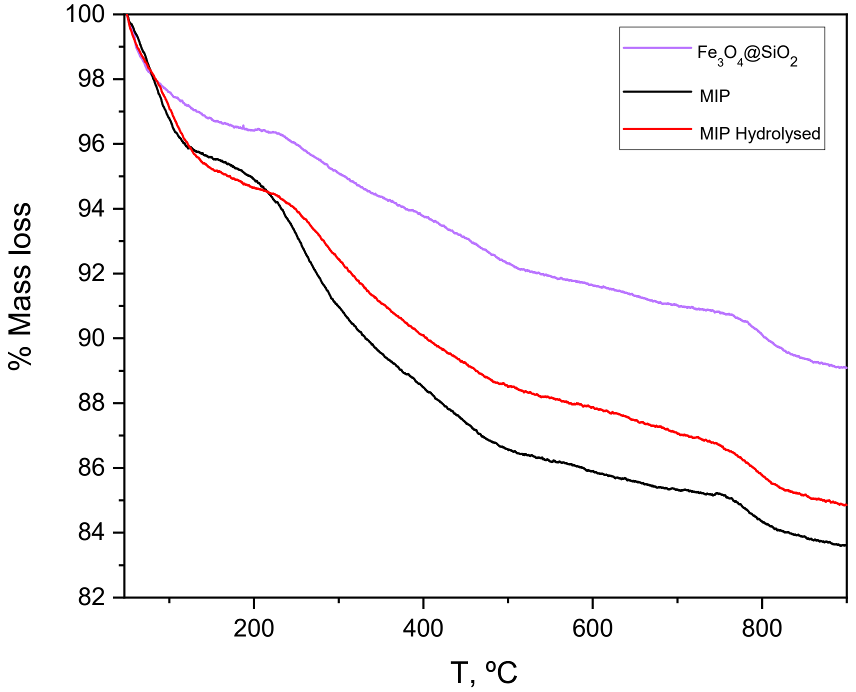

Figure S3. Thermogravimetric analysis (TGA) of $Fe_3O_4@SiO_2$ (purple line), MIP (black line) and MIP nanoparticles after hydrolysis of the template molecule (red line).

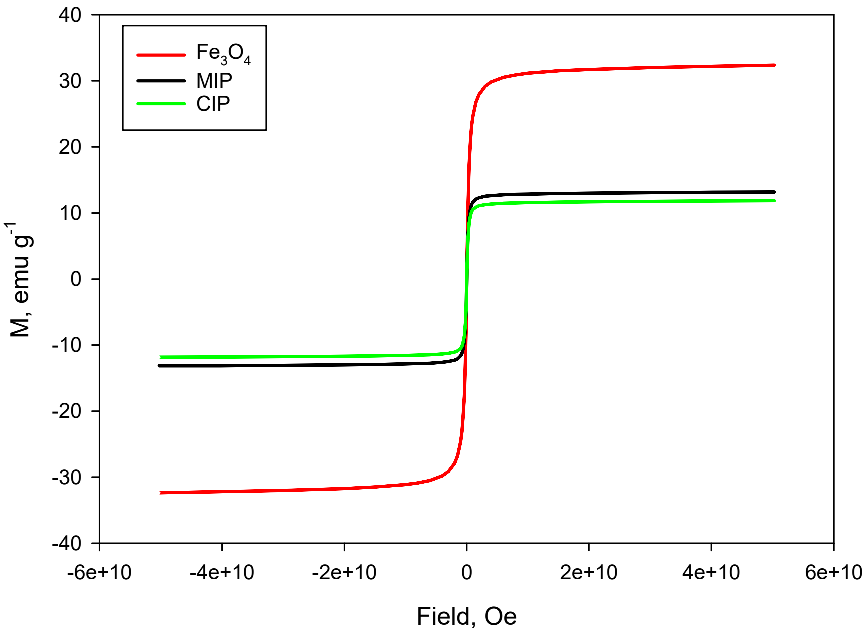

Figure S4. Magnetism study of Fe_3O_4 magnetic nanoparticles (red line), MIP (black line) and CIP (green line).

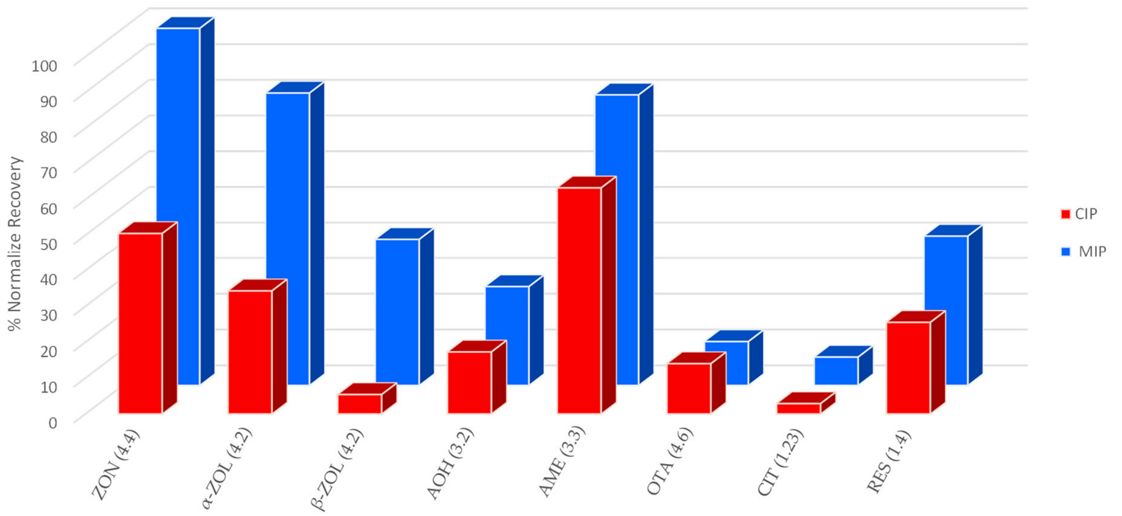

Figure S5. Recovery (%) of ZON, α-ZOL, β-ZOL, AOH, AME, OTA, CIT, RES and TeA (V =1 mL in phosphate buffer 50 mM, pH = 7.5) spiked at 150 μg L^{-1} with each of the tested compounds (RSD ≤ 22%, n = 3) and their corresponding logP values.

Table S1. Microanalysis of the MNPs before and after the coatings, MIPs and CIPs after the hydrolysis process for the extraction of the respective template molecule.

Sample	% C (±0.35)	% H (<LC)	% N (±0.30)
Fe_3O_4	2,99	0,73	0,13
Fe_3O_4@SiO_2	2,59	1,54	0,17
Fe_3O_4@SiO_2@MISG	12,76	1,93	0,93
Fe_3O_4@SiO_2@MISG@ hydrolysis	3,48	1,47	0,63
Fe_3O_4@SiO_2@CISG	14,39	2,59	0,79
Fe_3O_4@SiO_2@CISG@ hydrolysis	4,57	1,65	0,55
